# Evaluation of antiretroviral therapy (ART) provision in an early cohort of patients initiating ART in Ghana

**DOI:** 10.11604/pamj.2013.16.117.3136

**Published:** 2013-11-26

**Authors:** Sally-Ann Ohene, Nii Akwei Addo, Francisca Zigah, Morkor Newman, Margaret Lartey, Maite Alfonso Romero, Sampson Ofori, Tania Sheriff, Tom Ndanu

**Affiliations:** 1 World Health Organization Ghana Country Office, Accra, Ghana; 2 National AIDS/STI Control Programme, Ghana Health Service, Accra, Ghana; 3 Korle Bu Teaching Hospital, Accra, Ghana; 4 World Health Organization Inter-country Support Team, Harare, Zimbabwe; 5 Department of Medicine, University of Ghana Medical School, Accra, Ghana; 6 St Dominic's Hospital, Akwatia, Ghana; 7 Koforidua Regional Hospital, Koforidua, Ghana; 8 Odorna Clinic, Accra, Ghana; 9 University of Ghana Dental School, Accra, Ghana

**Keywords:** Ghana, antiretroviral treatment, immunological, clinical, outcomes

## Abstract

**Introduction:**

Against the background of Ghana's ART program which scaled up rapidly since inception in 2003, the study assessed outcomes of an early cohort of patients initiating ART.

**Methods:**

The study utilized the following methods: a cross-sectional study involving patient interviews using a structured questionnaire, a review of records and a retrospective cohort analysis of adults initiating ART between 2003 and 2008 from four selected clinics.

**Results:**

The 683 study participants consisted of 464 females (67.9%) and the mean age was 41 years. Mean duration of treatment was 25 months (SD =13). More than 95% were on a regimen as per national guidelines. Ninety-five (14.1%) of the respondents had one or two drugs substituted. Seventy-three% of the substitutions were due to adverse drug reactions. On at least one occasion, over half (350) had defaulted on a clinic appointment. In the 3 months preceding the survey, 21.4% (146) had missed treatment doses. About 49% (334) had challenges meeting financial obligations related to care. The median weight increased by 5.9kg and 8.0kg at 6 and 12 months after initiating ART respectively over the median baseline weight of 54kg, (p-value = 0.001). The median CD4 count increased by 128, 170 and 256 cells/µl respectively at 6, 12 and 24 months from the median baseline of 125 cell/µl, (p-value = 0.035).

**Conclusion:**

This study of Ghanaian PLHIV on ART from four facilities showed encouraging immunological and clinical outcomes. There were however issues of appointment default, sub-optimum adherence to treatment and cost of care barriers needing attention.

## Introduction

In consideration of the profound effects HIV has on the many people who are infected and affected particularly in sub-Saharan Africa, the World Health Organisation (WHO) in 2003 launched the “3 by 5” initiative which sought to make Anti-retroviral Therapy (ART) available to Persons Living with HIV (PLHIV) especially those in the developing world. [1] In tandem with this venture, Ghana embarked on its ART program in 2003. Counting from the 100 Ghanaian PLHIV who were first initiated on treatment in Ghana's ART pilot program in the first year, the number exceeded 10,000 by the end of 2007 and rose to over 47,000 by December 2010. [2, 3]

The introduction of highly active antiretroviral therapy (HAART) dramatically changed the course of HIV infection and has led to a significant reduction in HIV-related morbidity and mortality globally.[4] Several studies have documented long term outcomes among PLHIV on ART in sub-Saharan Africa demonstrating good immunologic, virologic, and clinical response to their treatment. [5–10] A number of these reports have highlighted various issues pertaining to life-long ART including the regimens used, adherence to treatment and adverse effects encountered providing valuable insight on the experience of PLHIV in the sub-region. In Ghana a limited number of studies have profiled various outcomes in PLHIV taking ART. [11–13] A study on short term outcomes among a sample of PLHIV initiated on treatment as Ghana scaled up ART was undertaken. We present these findings to fill in the gap in the picture of the history of ART provision in Ghana.

## Methods

The study consisted of a cross sectional study and a chart review. The participants in this study were recruited from four purposively selected levels of health facilities namely teaching, regional, district and private hospitals offering ART services in Ghana. Two sites were public, one private and one faith based institution. The sites were Korle Bu Teaching Hospital (KBTH), Koforidua Regional Hospital (KRH), the St Dominic's Hospital (SDH) and Odorna clinic, a private provider. All four facilities were selected for being pioneers in their range to provide ART and were using the prevailing national guidelines issued by the National AIDS/STI Control Programme. The guidelines stipulated WHO clinical stages 3 or 4, CD4 < 250 and a least 2 sessions of adherence counseling as requirements before initiation of ART.

First line therapy first choice drugs consisted of Zidovudine (AZT), Lamivudine (3TC) and Nevirapine (NVP or. Efavirenz (EFV). Stavudine was an alternative to Zidovudine. Routine laboratory tests, weight and CD4 count were measured at initiation. Weight was repeated at each clinic visit, laboratory tests at 3 months and CD4 tests 6 monthly. Patient data was recorded in both manual and electronic formats at each visit. PLHIV accessing ART were required expected to contribute a monthly fee equivalent to $5.00 towards clinical services but were not denied treatment if they could not pay.

### Inclusion Criteria

The study employed a retrospective cohort analysis of data from adult HIV positive patients18 years and above on ART. Eligible study participants consisted of those who had been on antiretroviral therapy for at least 6 months and were attending ART clinic for the first time in the four sites within the study period of June - August of 2008. During this time frame, consecutive patients attending the ART clinic, fitting into the inclusion criteria and who gave their consent were recruited into the study.

### Data collection and analysis

Chart reviews included socio-demographic data, WHO staging, weight and CD4 counts, drug regimens, adherence records, treatment default and clinical progress specifically new opportunistic infections developed while on treatment. Face to face interviews were utilized to corroborate chart findings and also explore drug related side effects, affordability of treatment and factors related to accessing ART services. The data collected was entered into Microsoft Access database version and consistency was checked for quality control purposes. SPSS software/programme was then used to conduct the analysis. Descriptive statistics were used to outline the demographic, treatment and clinical characteristics. Outcomes of interest were changes in CD4 count and body weight over baseline at 6 months intervals. To evaluate the significance of changes in CD4 and weight over time using repeated measures, the Friedman test for comparing repeated median values was used.

Ethical clearance for this study was obtained from the Ethical and Protocol Review Committee of the University of Ghana Medical School. Written informed consent was obtained from all study participants.

## Results

**Socio-Demographic Characteristics** Over the study period June to August 2008 a total of 683 respondents were recruited the majority of which were females (Table 1). Almost three quarters of the participants (496, 72.7%) fell within the age interval of 30 to 49 years. More than 80% (564) had at least primary education. Even though more than eight out of ten (568, 83.2%) of the respondents indicated they had children, most of those parents (70%, 398) did not know the HIV status of their children.

**Table 1:** Demographic and treatment characteristics of PLHIV who had been taking ART at least 6 months by the time of the study (N=683)

Number from study sites	n (%)
Korle Bu Teaching Hospital	408 (59.8)
Koforidua Regional Hospital	138 (20.2)
St Dominic's Hospital	104 (15.2)
Odorna Clinic	33 (4.8)
**Age, years**	
Mean (SD)	41.4 (9.2)
Range	18 – 72
Women, n (%)	464 (67.9)
**Educational status, n (%)**	
Nil	119 (17.4)
Primary	118 (17.3)
Junior secondary/middle school	285 (41.7)
Senior secondary/high school	116 (17.0)
Tertiary	38 (5.6)
Other	7 (1.0)
**Religion, n (%)**	
Christian	612 (89.6)
Moslem	60 (8.8)
Other	11 (1.4)
**Point of HIV diagnosis/ source referral to ART clinic, n (%)**	
Medical diagnostic testing	290 (42.5)
Pediatric diagnostic testing	14 (2.0)
Surgical diagnostic testing	8 (1.2)
TB clinic diagnostic testing	24 (3.5)
PMTCT / ANC	21 (3.1)
VCT	60 (8.8)
Partner HIV positive	48 (7.0)
Referral from other health Institutions	206 (30.2)
Others	12 (1.6)
**Initial ART regimen, n (%)**	
Zidovudine + Lamivudine + Nevirapine	244 (35.7)
Zidovudine + Lamivudine + Efavirenz	230 (33.7)
Stavudine + Lamivudine + Nevirapine	103 (15.1)
Stavudine + Lamivudine + Efavirenz	78 (11.4)
Other combinations	28 (4.1)
**Duration on ART, n (%)**	
6 – 12 months	198 (28.0)
13 – 24 months	218 (32.0)
25 – 36 months	145 (21.2)
≥37 months	122 (18)
**Means of paying ART services, n (%)**	
Patient out of pocket	456 (66.8)
Family	87 (12.7)
Spouse	46 (6.7)
Employee sponsored	7 (1.0)
Medical insurance	4 (0.6)
Other	83 (12.2)
**Times drug collected without making payment, n (%)**	
Never	179 (26.6)
1-4 times	293 (43.5)
5-9 times	136 (20)
>10 times	64 (9.5)
No response	11(1.6)

### Antiretroviral Therapy

**Knowledge of HIV status and travel time to clinic** Enquiring about how participants found out their HIV diagnosis and subsequently referred to the ART clinic revealed that 45.7% were through diagnostic HIV tests for a medical or surgical condition while 7% had had HIV testing after their partner tested positive for HIV. For 42.8% travelling time to the clinic was in the range of 30 minutes to an hour. A quarter spent 1 to 2 hours getting to the clinic whiles 13.7% usually travelled more than 2 hours to get to their treatment sites.

### ART regimens

All the respondents had been taking ARVs for 6 months or more, with the mean duration of treatment being 25.15 months (SD =13.04). The longest period a participant had been on treatment was 59 months. Before initiating treatment, most people (90.7%) had undergone at least 2 adherence counselling sessions. Almost all the respondents (95.1%) were on a recommended first line regimen. More than two-thirds of these (69.4%) were on a regimen consisting of Zidovudine and Lamivudine with either Nevirapine or Efavirenz. combination. During the course of ARV therapy, 95 (14.1%) of the respondents had one or two of the ARVs medication substituted. Substitutions were made for the following numbers (proportions) of person on the respective ARVs as follows: Stavudine 20 (11%), AZT 35 (7.3%), NVP 25 (7.2%) and Efavirenz 15 (4.9%). For seven out of ten of the substitutions (73%), the reason was due to adverse drug reaction. For 13% it was due to initiating TB treatment. Three percent of the substitutions were as a result of pregnancy.

### Treatment and appointment default

About half, 350 (51.2%), of the respondents admitted to defaulting on their clinic appointments but a review of the medical charts showed that 77.7% of the respondents had defaulted on their appointment on at least one occasion. Reasons cited for defaulting varied from financial/ economical difficulties to travelling out of town and forgetfulness (Table 2). “Other reasons” included a myriad of responses such as delayed laboratory results, having to attend to a sick relative and mix up of clinic dates. Adherence to ARVs within the 3 months before the survey as per patient self report was assessed. Out of 146 respondents (21.4% of study population) who admitted defaulting on their treatment in the last 3 months, 118 (80.8%) had missed taking their drugs between 1 to 4 times and the rest missed their doses 5 or more times. Reasons given for the non-adherence to ARV varied from forgetfulness to running out of drugs (Table 2). Ten respondents (1.5% of those surveyed) said they stopped taking the ARVs entirely because of unpleasant effects including weakness and gastro-intestinal side effects.

**Table 2:** Reasons given by PLHIV taking ART for defaulting on clinic appointment and missing doses

Reasons given by PLHIV for defaulting on clinic appointment least once (N = 352)	Number (%)
Economic reasons	110 (31.3)
Travelled	68 (19.3)
Forgetfulness	41 (11.6)
Still had some drugs	33 (9.4)
Felt unwell	13 (3.7)
Other	88 (24.7)
	
**Reasons given by PLHIV for missing their doses at least once in the preceding 3 months (N = 146)**	Number (%)
Forgetfulness	66 (45.2)
Travelled without them	31 (21.2)
Ran out of drugs	27 (18.4)
Unpleasant side effects	8 (5.5)
Problems related to meals	7 (4.8)
Other	11 (7.5)

### Payment for Treatment

At the time of the study, as a national policy, PLHIV were charged Gh ¢ 5 (approximately US$5) monthly for as contribution towards cost of care for HIV management. This amount was usually collected at the pharmacy at the point of drug pick up. Enrolment in National Health Insurance Scheme (NHIS) did not exempt a person from paying this fee so even the 53% of respondents who reported being enrolled in the NHIS were still expected to pay the service charge. Patients who could not pay however because of inability to afford the fee or did not have money available on the clinic day were still entitled to pick up their drugs. About two-thirds of participants stated that they paid for their treatment out of pocket. More than 7 out of ten respondents however indicated that on at least one occasion they had collected their drugs without paying the required fee (Table 1). About half of the study participants (334) had an outstanding bill to pay at the time of being interviewed for the study.

### Opportunistic infections and tuberculosis

Opportunistic infections (OI)/conditions that the study population had been managed for while on ART were recorded from the patient charts. At least one episode of OI was recorded for 460 out of 683 participants (67%). The number treated for consecutive second, third, fourth and fifth episodes of OI whiles on ARVs were 276 (40%), 169 (25%), 103 (15%) and 64 (9%) respectively. The three commonly reported conditions managed for each episode were respiratory disorders, malaria and skin lesions. These three accounted for over 70% of the opportunistic infections/ conditions reported in each episode. A history of being investigated for TB was elicited by checking for assessment of sputum smears and or chest x-rays from medical chart reviews and collaboration from respondent interviews. It was found that 42% (286) of the respondents had been investigated for TB. Of the 267 who had undergone sputum smear testing, 22.8% (61) had a smear positive sputum for AFB while 28.5% (71) of the 249 who had had chest X-ray taken had findings suggestive of TB.

### Treatment outcomes

**Figure 1:**
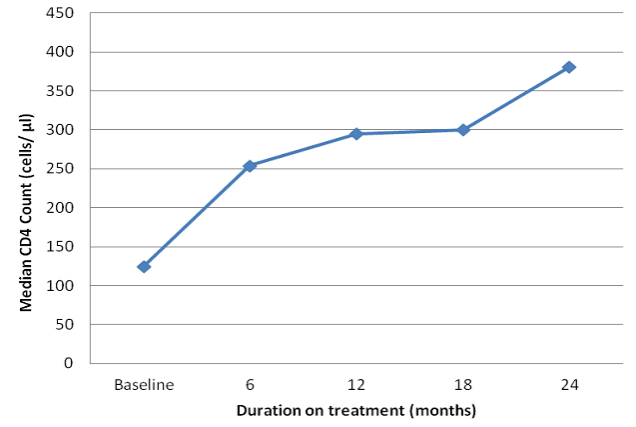
Changes in median CD4 count of patients on ART from baseline at six monthly intervals

After the initiation of ART, changes in CD4 counts over the baseline levels were analyzed at 6 month interval using Freidman's Test for repeated measures to assess significance in the changes. Due to the different starting points of ART for the study participants and unavailability of data in some cases, the number of those who had CD4 count available for analysis decreased over time from 579 at baseline to 200 after 12 months and 183 after 24 months on treatment. The median CD4 count increased by 128, 170 and 256 cells per microliter (µl) respectively at 6, 12 and 24 months over the median baseline of 125 cell/µl (p = 0.035). Figure 1 shows the median CD4 counts at the various intervals for the specified numbers of participants whose CD4 counts were available for analysis. Similarly an assessment of changes in the median weight increase over the baseline was tracked again using Freidman's Test for repeated measures to assess whether the differences were significant. The number of participants for whom weight was available for analysis at baseline, 6 months and 12 months was 528, 293 and 156. Increases of 5.9 kg and 8.0 kg over the median baseline weight of 54 kg (p = 0.001) at 6 and 12 months after initiating ART respectively were noted. Thereafter, there was stabilization of the median weight among the relatively fewer number of patients for whom weight data was available.

## Discussion

This study on the experience of an early cohort of PLHIV initiated on ART during the scale up of Ghana's ART program points to significant improvement in weight and CD4 counts over the course of their treatment. Almost all the participants had been initiated on the first line drug regimen recommended in the national guidelines. More than a fifth however admitted to missing their drugs on at least one occasion in the three months preceding the survey. About half had been unable to honour their contribution to the cost of care. The ability of the study to bring out these findings as well as other characteristic related to initiating and taking ART including issues pertaining to entry to care, drug substitutions clinic attendance and the common opportunistic conditions encountered while on ART highlights its strength. As such, the study contributes to the available literature by providing the missing piece of data on the early years of ART in Ghana. This is in spite of the following study limitations: dependence on routine clinic records and patient reports with the associated problems of missing data and recall bias respectively and the exclusion of those on treatment who had been transferred out or were lost to follow up at the time of the study.

The study participants comprised of more women than men, similar to the situation in most sub-Saharan countries [5, 6, 15] and also reflective of the national proportion of males and females receiving ART [16]. The age ranges and the educational level were found to be comparable with that from a study of PLHIV on ART in Kumasi, another city in Ghana. [13]. It was surprising to note that among those who had children, the HIV status of two-thirds of the children was not known by the study participants. Considering the possibility of vertical or horizontal transmission, every opportunity should be taken to recommend HIV testing and counselling for family members, especially young children, to facilitate treatment and care for those who might need them. [17]

As antiretroviral therapy is scaled-up in Ghana, there should be a push for early identification of persons with HIV infection so they can benefit from the services provided. The study showed that diagnostic testing was the major source of patient identification. This is not too surprising since all our study participants were on ART indicating they were at a late stage of HIV infection to warrant treatment. It is possible that they underwent diagnostic testing because they presented at a health facility with a Stage 3 or 4 disease. In their report on the outcomes of PLHIV initiated on ART in the early part of Botswana's National ART Program, Bussman and colleagues also highlighted the point that a large proportion of their patients showed evidence of advanced stages of HIV infection. [6] To reduce late initiation of ART for better outcomes however it is prudent for PLHIV to be diagnosed early in the infection. [5, 18] Thus efforts at improving access to counselling and testing (CT) such as offering outreach CT services at the community level and provider initiated routine offer of CT should be actively pursued and scale up [17].

It is reassuring to note that practically everyone had the minimum number of 2 adherence counselling sessions as recommended in the ART guidelines. [14] Also gratifying is the fact that the recommended combinations of first line ART for treatment were being prescribed to over 95% of the patients in sync with what was found for attendees in another major ART clinic in the Ghana program.[13]

The proportion of respondents on respective ARVs who had their drugs substituted is comparable to what others have found. [12, 19] Most of the substitutions in our study participants were because of adverse events. Other researchers also cite these reasons among the significant reasons for substitution. [12, 19–21] Gastro-intestinal symptoms were among the unpleasant effects reported by our study respondents who stopped taking their ARV entirely. This concurs with the study by O'Brien and colleagues who also report that gastrointestinal adverse events of ARVs were the most frequently cited reason for discontinuation of treatment.[22] It is therefore imperative for health staff caring for PLHIV on ART to regularly ask clients about adverse drug reactions and side effects to facilitate appropriate management according to national guidelines.

From the study about half of the respondents admitted they had defaulted on their treatment appointment but the review of the medical chart revealed a much greater proportion had defaulted. Other studies have reported a relatively lower appointment default rate probably due to the differences in the definitions used which in our case was missing at least one appointment. [23, 24] It is important to note that successful treatment with highly active antiretroviral therapy (HAART) requires the patient to maintain consistent adherence to the prescribed regimen and keeping of clinic appointments enable patients come for follow up and refill prescriptions. [25] So defaulting on appointments poses a risk to treatment adherence and steps have to be taken to address barriers. The reasons our study participants gave for defaulting have been corroborated in multiple studies. [25] Similarly the proportion of those who missed taking their drugs and the reasons they gave for failing to do so are comparable to other Ghanaian PLHIV on ART. [13] To facilitate adherence it is key to adopt effective adherence support interventions which include treatment supporters, patient centred counselling and addressing barriers such as cost.[17, 26] This perhaps buttresses the need for having an adherence monitor as stated in the ART guidelines since the monitors are expected to help patients adhere to treatment through reminders and other means. It is also important to look at the role played by economic constraints on appointment defaults and therefore running out of drugs. Two-thirds of the study participants said they paid for their care out of pocket and about half had not kept up with the payment of the monthly fee and owed various amounts at the clinic. Much as in principle clients are not denied treatment because of inability to pay, it is possible that embarrassment at owing money may deter some people from showing up at the clinic. [13] There is a need to reconsider the issue of payment for HIV care as it may have implication for treatment adherence.

The opportunistic infections/conditions reported in our study are among those commonly reported in PLHIV. [27–29] Over a fifth of our participants investigated for TB were found to have a positive results reiterating the point that PLHIV are still very much at risk for contracting TB even though they may have initiated ART. [27] Our study findings show that the study participants responded well to treatment over time. Immune recovery evident by increasing CD4 and weight gain was progressive in the course of the year of starting treatment. These findings are in keeping with what is expected when immunity builds up and HIV infection is controlled in a PLHIV on treatment. [5, 6, 30] These desired outcomes are made possible when clients are managed according to national guidelines, barriers to taking treatment are addressed and treatment is taken as prescribed.[17] As Ghana continues to scale up and aims for universal access to HIV prevention, treatment and care services, it is encouraging to note that those enrolled in the program are making good progress. It is however imperative that the follow up of those on treatment is maintained to address issues related to taking their treatment including adverse drug reactions. This will contribute to PLHIV who are effectively managed on ART living productive lives and minimize the emergence of HIV drug resistance.[17] Building on this study, further analysis of long term outcomes is warranted to assess whether the clinical and immunological outcomes are maintained. Future studies should also assess viral suppression in response to treatment.

## Conclusion

In summary, this study of an early cohort of PLHIV from the four treatment facilities initiated on ART during the scale up of ART showed encouraging outcomes immunologically and clinically. HIV care management was as per national guidelines. There were however issues of defaulting on appointment, sub-optimum adherence to treatment and cost of care barriers needing attention. To maximise the benefits of ART, it is imperative to maintain regular follow up of PLHIV on treatment and ensure appropriate clinical and immunological monitoring. Information on drug effects should be regularly elicited from PLHIV to facilitate prompt and optimum management. This will enable those not achieving the desired outcomes to be identified for effective management measures to be taken. It is recommended that clinicians and policy makers take note of the findings from this study to inform management of PLHIV and planning as even more people are enrolled on treatment to ensure quality of care of the ART program.
